# Comparison of methods to normalize urine output in critically ill patients: a multicenter cohort study

**DOI:** 10.1186/s13054-024-05200-x

**Published:** 2024-12-19

**Authors:** Céline Monard, Nicolas Tebib, Bastien Trächsel, Tatiana Kelevina, Antoine Guillaume Schneider

**Affiliations:** 1 Adult Intensive Care Unit, Centre Hospitalier Universitaire Vaudois, University Hospital of Lausanne, 1011 Lausanne, Switzerland; 2 Department of Epidemiology and Health Systems, Center for Primary Care and Public Health (Unisanté), University of Lausanne, Lausanne, Switzerland; 3 Faculty of Biology and Medicine, University of Lausanne, Lausanne, Switzerland

**Keywords:** Definition, Oliguria, Urine output, Weight

## Abstract

**Background:**

Oliguria diagnosis includes the normalization of urine output (UO) by body weight. However, the rational and the method to apply to normalize UO to body weight are unclear. We aimed to explore the impact of the method applied to normalize UO on oliguria incidence and association with outcomes.

**Methods:**

We included all adult patients admitted to a Swiss (derivation cohort) and a US (MIMIC-IV database, validation cohort) ICU, except those on maintenance hemodialysis, who declined consent or had < 6 consecutive UO measurements. Among a panel of candidate variables (ideal body weight, body mass index, body surface area and adjusted body weight), we identified the best predictor for UO (*i.e.* the variable that was most closely associated with mean UO during ICU stay). We then compared oliguria incidence and association with 90-day mortality and acute kidney disease (AKD) at hospital discharge, according to whether UO was normalized by actual body weight (ABW) or the identified best UO predictor.

**Results:**

The derivation and validation cohorts included respectively 15 322 and 28 610 patients. Those in the validation cohort were heavier (mean ABW 81 versus 75 kg) older (65 versus 62 years) and had a lower SAPS-II score (38 versus 43). The best UO predictor was ideal body weight (IBW). Oliguria incidence increased almost linearly across weight categories with ABW normalization but remained constant with IBW normalization. Using IBW for UO normalization rather than ABW improved the association between oliguria and 90-day mortality and AKD. It increased the proportion of patients correctly classified from 37.6 to 48.3% (mortality) and from 37.8 to 47% (AKD). All findings persisted after correction for sex and SAPS-II score and were confirmed in sensitivity analyses.

**Conclusion:**

UO normalization by IBW lead to a stable incidence of oliguria across categories of weight and improved the association between oliguria and outcomes. IBW should be preferred to normalize UO in critically ill patients.

**Supplementary Information:**

The online version contains supplementary material available at 10.1186/s13054-024-05200-x.

## Introduction

According to the kidney disease: improving global outcomes (KDIGO) consensus, oliguria is defined as a urine output (UO) of less than 0.5 ml/kg/h for 6 h or more [1]. Based on this definition, oliguria is observed in up to 75% of critically ill patients [2, 3] and associated with 90-day mortality, irrespective of changes in serum creatinine (sCr) [2, 4]. However, the rational and the method to apply to normalize UO to body weight (BW) are unclear. Indeed, in critically ill patients, pre-admission BW might be difficult to retrieve, particularly in uncounscious patients, and its estimation by nurses has been shown to be highly inaccurate [5, 6]. In addition, the actual body weight (ABW) is subject to massive variations, mostly related to fluid overload and, to a lesser degree, muscle mass loss [7]. Finally, several *types* of BW can be considered: pre-admission, actual, ideal or adjusted BW. These might significantly differ, particularly in obese or underweight patients. The lack of standardization of the considered BW to normalize UO might greatly influence the observed incidence of oliguria and thus AKI, and its association with outcomes.

To the best of our knowledge, only a handful of studies have addressed this issue. In a series of 493 patients, normalization of UO by the *actual* BW (ABW) led to a higher observed incidence of oliguria than normalization by the *ideal* BW (IBW). Oliguria was significantly associated with mortality only when normalized by IBW [8]. These results were confirmed in two larger studies, conducted in the United States and Germany [9, 10]. In a fourth study, conducted in 569 patients with sepsis, the association between oliguria and mortality was not influenced by the method used to normalize UO [11]. However, none of these studies explored the relationship between weight, height and UO during the ICU stay nor have they compared several candidates for UO normalization in a large population of critically ill patients. In addition, they were all single center studies and lacked validation in a cohort with different weight distributions. Hence, to date, no definitive conclusions can be made on how to normalize UO for oliguria and AKI diagnosis.

Accordingly, we sought to leverage two large datasets from two different countries and health care systems to explore the impact of the method applied to normalize UO on the observed incidence of oliguria and its association with outcomes.

## Methods

### Study population

This study used data from two cohorts, the previously described Laus’AKI cohort (as derivation cohort) and the Medical Information Mart for Intensive Care IV (MIMIC-IV) cohort (as validation cohort) [2, 12, 13]. For both cohorts, only the first eligible ICU admission was considered.

#### Laus’AKI cohort (derivation cohort)

The Laus’AKI cohort included all adult (≥ 18 years) patients admitted within the multidisciplinary ICU in Lausanne, Switzerland between January 2010 and June 2020. We excluded patients who refused participation, were receiving maintenance hemodialysis, had less than 6 h of UO measurement or no sCr available [2]. The dataset includes data from electronic medical records (Metavision^®^, iMD Soft, Tel Aviv, Israel), and Soarian^®^ (Cerner, North Kansas City, USA)) as well as the Swiss national death registry.

#### MIMIC-IV cohort (validation cohort)

MIMIC-IV is an open dataset including all ICU admissions that occurred in the Beth Israel Deaconess Medical Center (Boston, United States of America) between 2008 and 2019 [12, 14]. It includes data obtained from electronic medical records (Metavision®, iMD Soft, Tel Aviv, Israel), the hospital data warehouse and external data sources including international classification of diseases (ICD) codes and out-of-hospital mortality. We applied to the MIMIC-IV dataset, the same inclusion/exclusion criteria applied to the Laus’AKI cohort (see above). In addition, we excluded patients with missing data for weight or height and those who received vesical irrigation during their ICU stay as this could not be distinguished from urine.

### Data extraction

For both cohorts, we extracted patient's characteristics, severity scores, admission status (elective or emergency), ICU diagnoses and interventions, ICU and hospital length of stay as well as hospital and 90-day mortality. We also retrieved all sCr measurements obtained while in hospital as well as hourly UO measurements obtained while in ICU. For missing hourly UO values, we assigned values (prior to normalization) to each empty hour by distributing evenly the next available value across missing hours (e.g., the sequence [“NA”, “NA”, “NA”, 200] was replaced with [50, 50, 50, 50]). Differences in data management between the two cohorts as well as missing values management are described in Table S1.

### Oliguria assessment

In both cohorts, we retrieved hourly UO measurements and, for each hour, calculated a 6-h mean corresponding to the mean UO measured over the preceding 6 h. According to the KDIGO definition, patients were considered to have presented oliguria if any 6-h mean value was < 0.5 ml/kg/h [1].

### Definitions

In the Laus’AKI cohort, the reference body weight was the pre-admission actual body weight (ABW) collected from medical records. If no pre-admission ABW was available, we considered the first quartile of all body weights measured while in ICU. When no weight was measured, we attributed a weight of 60 kg to women and 70 kg to men. Details on weight selection and missing variables are reported in Table S1.

Acute kidney disease (AKD) at hospital discharge was defined as a 35% decrease in discharge eGFR compared to baseline eGFR, or a 150% increase in discharge sCr compared to baseline. To compute baseline and discharge eGFR we used the CKD-EPI 2021 formula with either the baseline sCr (baseline eGFR) or the closest sCr to hospital discharge (discharge eGFR). The selection of the considered baseline sCr is described in Table S1.

Simplified acute physiology score (SAPS) II was available in both datasets. For the purpose of these analyses, this score was corrected not to include the UO component.

### Statistical analyses

First, to identify the best predictor of UO during the ICU stay, we examined the association between different candidate variables and mean 6-h average UO in the Laus’AKI cohort. As candidate variables, in addition to the ABW, we considered: the *ideal* BW (IBW), a function of patient’s height, using several classic equations (Devine (IBW_d_), Peterson, Hammond and Miller) [17–19], the *adjusted* body weight (AdjBW) according to the following formula: AdjBW = IBW_d_ + 0.4*(ABW- IBW_d_), the body mass index, the patient’s height and the body surface area using the Mosteller, Dubois and Haycock equations [15–17]. Formulas used to calculate these candidate variables are presented in Table S2. We compared the strength of these associations by examining the Akaike’s Information Criteria (AIC) and R-squared (R^2^) of the different regression models. We used generalized linear models including the considered variable modeled using spline [18]. Analyses were repeated using minimum and maximum 6-h average UO for confirmation. The candidate variable with the lowest AIC and highest R^2^, was retained for further analyses (“best UO predictor”).

We then compared the *incidence* of oliguria in the derivation and validation cohorts according to whether UO was normalized by ABW or the best UO predictor. These data were stratified by groups of 10 kg of ABW.

Then, in the MIMIC-IV cohort, we applied logistic regression to assess the strength of the association between oliguria and outcomes (90-day mortality and AKD) accounting for sex and corrected SAPS-II. These analyses were performed after UO normalization by (1) ABW and (2) the best UO predictor. The performance of both methods was compared using respective model’s area under the receiver operator curve (AU-ROC) to estimate its discrimination power, and the McFadden’s adjusted R^2^ (pseudo R^2^) to estimate its predictive power [19]. The pseudo R^2^ represents the percentage of variation explained by the model; a pseudo R^2^ of 100 would mean that the model perfectly predicts all deaths, while a pseudo R^2^ of 0 would mean that the model is not useful to predicts deaths.

In the MIMIC-IV cohort, we then compared the agreement between the maximum AKI stage observed using sCr or UO criteria according to whether UO was normalized by ABW or the best UO predictor. We assessed the proportion of patients achieving similar stage and assessed their correlation using Kendall rank correlation coefficient.

Finally, we performed several sensitivity analyses in the validation cohort. For these, we repeated our main analyses after exclusion of (1) patients who did not have an indwelling catheter throughout their ICU admission and (2) patients who received diuretics while in ICU.

Continuous data are reported as mean (standard deviation, SD) or median (interquartile range, IQR) and categorical variables expressed as number (percentage). For all analyses, a two-tailed p-value < 0.05 was considered as statistically significant. Statistical analyses were performed with R® (R Core Team, Auckland, New Zealand, including the following extension packages: data.table, pROC, sjPlot, DescTools, splines [20–24]).

This report follows “The Strengthening the Reporting of Observational Studies in Epidemiology” (STROBE) guidelines for reporting the results of this study.

### Ethics

The Laus’AKI cohort was approved by the Ethics Committee of canton de Vaud (CER-VD 2017–00008, Lausanne, Switzerland). In adherence to the Swiss Federal Act on Research involving Human Beings (article 34), retrospective utilization of non-genetic health-related personal data was allowed, provided that the patient (or its legal representative) had not expressed wishes of non-participating to clinical research.

Regarding MIMIC-IV, the Institutional Review Board at the Beth Israel Deaconess Medical Center granted a waiver of informed consent and approved the sharing of the research resource. Authors involved in data analyses completed a training in human research and signed a data use agreement.

## Results

### Patients’ characteristics

The datasets included respectively 21 668 (Laus’AKI) and 71 111 (MIMIC-IV) ICU admissions. Of those, 15 322 (Laus’AKI, derivation cohort) and 28 591 (MIMC-IV, validation cohort) were included in the study. Most (31 946, 75.1%) exclusions in MIMIC-IV were related to incomplete ABW or height data (details of exclusions in Fig. S1).

Patients’ baseline characteristics are presented in Table 1. Compared with those included in the Laus’AKI cohort, patients in the MIMIC-IV cohort were older (65.0 versus 62.4 years), had a higher mean ABW (82.7 versus 75.2 kg), a lower SAPS-II score (37.7 versus 43.3) and a lower proportion of them had an elective admission (19.2 versus 29.7%). In the Laus’AKI cohort, height was missing for 2 910 (19%) patients. Pre admission body weight was documented in 12 169 (79%) patients, in the rest, we used the first quartile of measured BW in 650 (4%), and attributed a BW based on sex to the remaining 2 503 (16%).

**Table 1:** Patient’s characteristics and outcomes

	Laus’AKI (Derivation cohort) (N = 15 322)	MIMIC IV (Validation cohort) (N = 28 591)
Age at ICU admission (years)	62.4 [16.2]	63.7 (16.1)
Sex (males)	10 156 (66.3)	17 100 (59.8)
Height (cm)	170.8 (8.3)	169.5 (10.8)
Body weight (kg)	75.2 (16.6)	82.7 (23)
Body mass index > 25 kg /m^2^	5 800 (46.7)	19 298 (67.5)
Body mass index > 30 kg /m^2^	2 090 (16.8)	9 898 (34.6)
SAPS II score at ICU admission (corrected)	43.3 (19.4)	36.7 (14.0)
Scheduled admission	4 444 (29.7)	5 442 (19.0)
Charlson comorbidity index	4.5 (3.1)	4.7 (2.9)
Chronic kidney disease	1 589 (10.4)	3 706 (13.0)
Baseline serum creatinine (mg/dl)	0.94 (0.7)	1.06 (0.3)
Diabetes	2 887 (18.9)	8 447 (29.5)
Hypertension	7 163 (46.9)	18 486 (64.7)
Diuretics administration during ICU stay	5 519 (36)	15 914 (55.7)
RRT during ICU stay	1 146 (7.5)	1 055 (3.7)
Type of UO measurement		
Spontaneous voiding (throughout stay)	1 472 (9.6)	3 903 (13.7)
Indwelling catheter (throughout stay)	11 898 (77.7)	17 284 (60.5)
Both	1 952 (12.7)	7 404 (25.9)
Outcomes		
ICU median length-of-stay (IQR)	2.25 (16.5)	2.29 (3.8)
Hospital median length-of-stay (IQR)	12.78 (4.5)	7.55 (8.1)
Acute kidney disease at hospital discharge^a^	1 913 (12.8)	5 541 (19.4)
ICU mortality	1 723 (11.2)	2 537 (8.9)
Hospital mortality	2 306 (15.1)	3 128 (10.9)
90-day mortality	2 859 (18.7)	5 225 (18.3)
Oliguria during ICU stay^b^		
Oliguria stage 1, UO normalized by ABW	10 989 (71.7)	21 833 (76.4)
Oliguria stage 1, UO normalized by IBW_d_	8229 (66.3)	18,307 (64.0)
Oliguria stage 2, UO normalized by ABW	8 418 (56.3)	16 471 (58.9)
Oliguria stage 3, UO normalized by ABW	2 130 (17.4)	4 832 (19.4)

Data are presented as mean (Standard Deviation -SD) for continuous measures unless otherwise indicated, and n (%) for categorical measures

ICU: intensive care unit; sCr: serum creatinine; AKI: acute kidney injury; RRT: Renal replacement therapy; ABW: actual body weight; IBW_D_: ideal body weight calculated by the Devine formula; Simplified Acute Physiology Score (SAPS)-II corrected does not include the points for urine output; UO: Urine Ouput

^a^Acute Kidney Disease at discharge is defined by a decrease in discharge eGFR > 35% compared to baseline eGFR or a discharge serum creatine > 1.5*baseline serum creatinine

^b^Oliguria stage 1 is defined by a urine ouput < 0.5 ml/kg/h for 6 h; oliguria stage 2 is defined by a urine ouput < 0.5 ml/kg/h for 12 h; oliguria stage 3 is defined by a urine ouput < 0.3 ml/kg/h for 24 h or anuria for 12 h

### Best predictor of UO during ICU stay

**Fig. 1:**
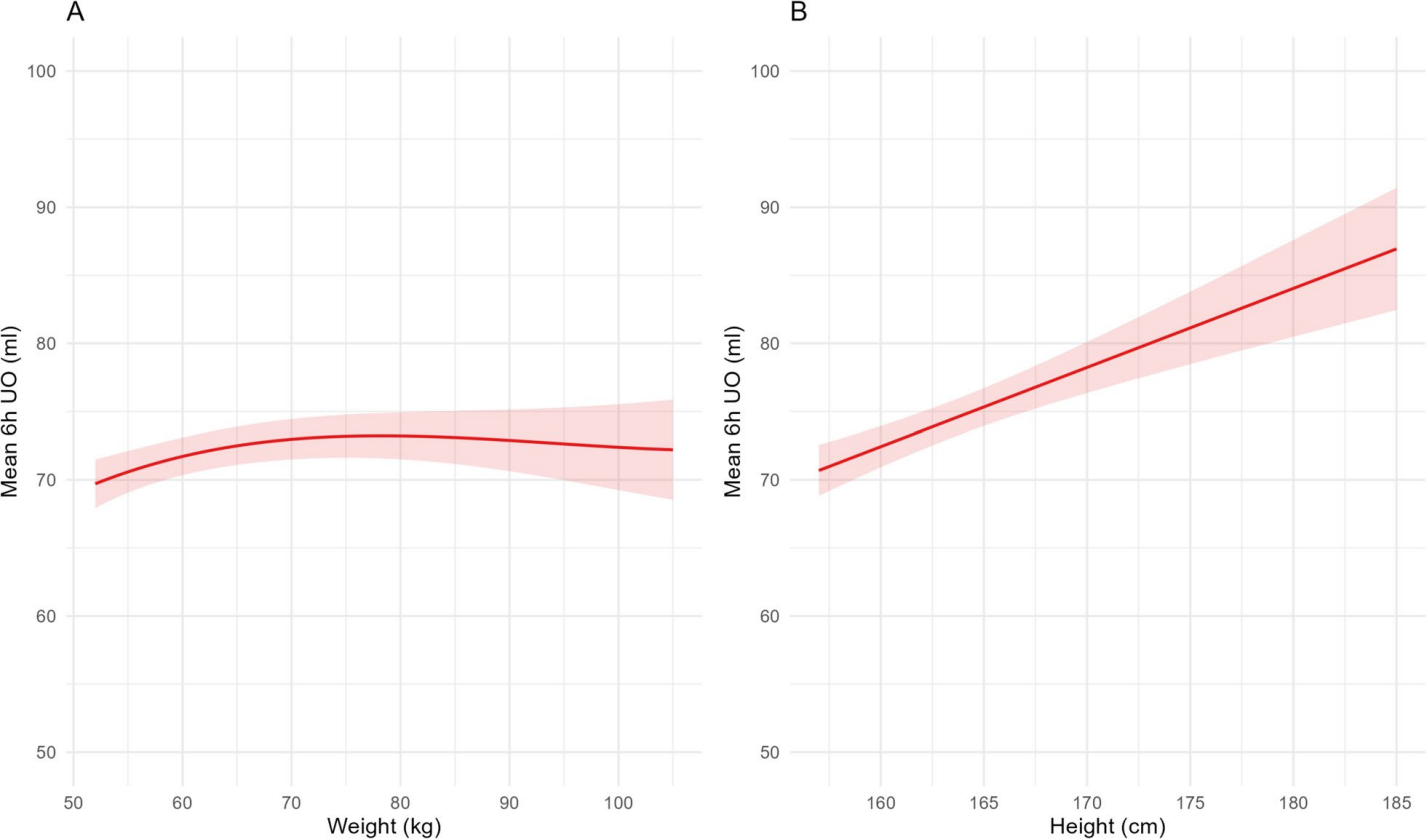
Relationship between mean 6-h urine output (UO) during ICU stay and actual body weight (panel a) or height (panel b) in critically ill patients. Values are mean (with 95% confidence intervals), n = 15 322 (panel A) and 12 412 (panel B)

As shown in Fig. 1, mean UO during ICU stay appeared to be more closely associated with patients’ *height* than to their weight. This association was also observed when minimal and maximal UO were considered (Figs. S2 and S3). Globally, candidate variables based on *height* exclusively (IBWs) performed the best to predict mean 6-h UO during stay. They were equivalent to each other’s and all superior to ABW (Table S3). Among those, since it is commonly used in clinical practice, we retained IBW calculated with the Devine formula (IBW_d_) as our “best UO predictor” for further analyses.

### Observed incidence of oliguria according to standardization method

**Fig. 2:**
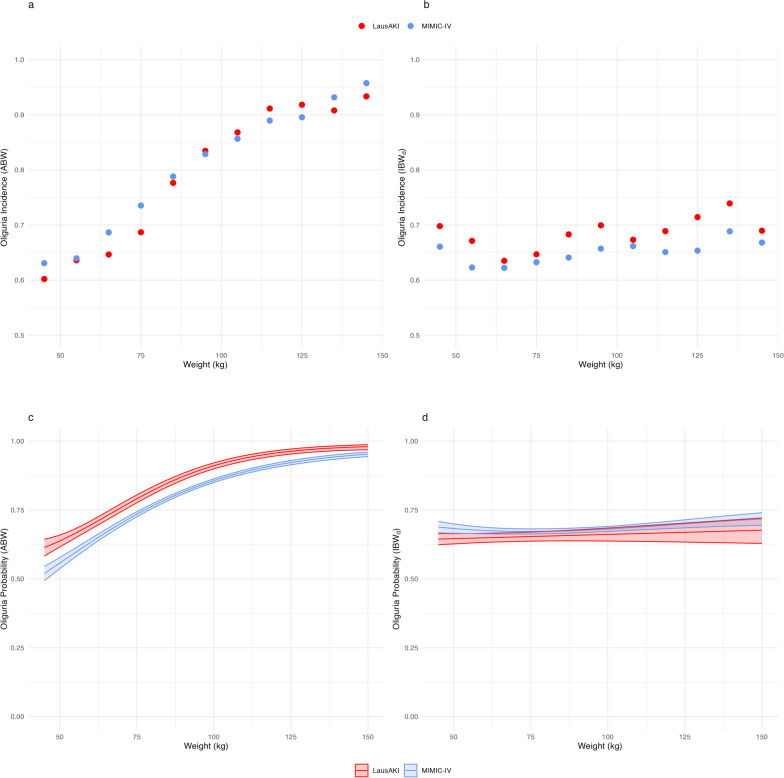
Observed incidence and probability of oliguria across weight categories according to UO normalization method. Upper panels: Observed incidence of oliguria across body weight categories when UO is normalized by ABW (panel A) or IBW_d_ (panel B). Lower panels: Probability of oliguria (with 95% confidence intervals) across body weight categories accounting for sex, and corrected SAPS-II score, when UO is normalized by ABW (panel C) or IBW_d_ (panel D). Results obtained in the validation and derivation cohort are represented in red and in blue respectively. ABW: actual body weight; IBW_d_: ideal body weight; UO: urine output. In the validation cohort, n = 15 322 (panels A and C) and 12 412 (panel B and D). In the derivation cohort, n = 28 591 (all panels)

The overall incidence of oliguria was slightly higher when UO was normalized by ABW compared with IBW_d_ (71.7 versus 66.3%). More importantly, there was an almost linear increase in the incidence of oliguria across categories of BW, with virtually all patients > 120 kg fulfilling criteria for oliguria when UO was normalized by ABW (Fig. 2, panel A). This persisted after adjustment for sex and SAPS-II score (Fig. 2, panel C). This means, for example, that, when ABW is utilized for UO normalization, the probability of fulfilling oliguria criteria for a 65-year-old man with a SAPS-II score of 35 would be 81% if his ABW is 87 kg versus 66% if it is 62 kg.

On the other hand, when UO was normalized by IBW_d_, the incidence of oliguria remained stable across categories of ABW (Fig. 2, panel B). This remained after adjustement for sex and SAPS-II score (Fig. 2, panel D). Findings were similar in both the derivation and the validation cohorts.

### Association of oliguria with outcomes

**Fig. 3:**
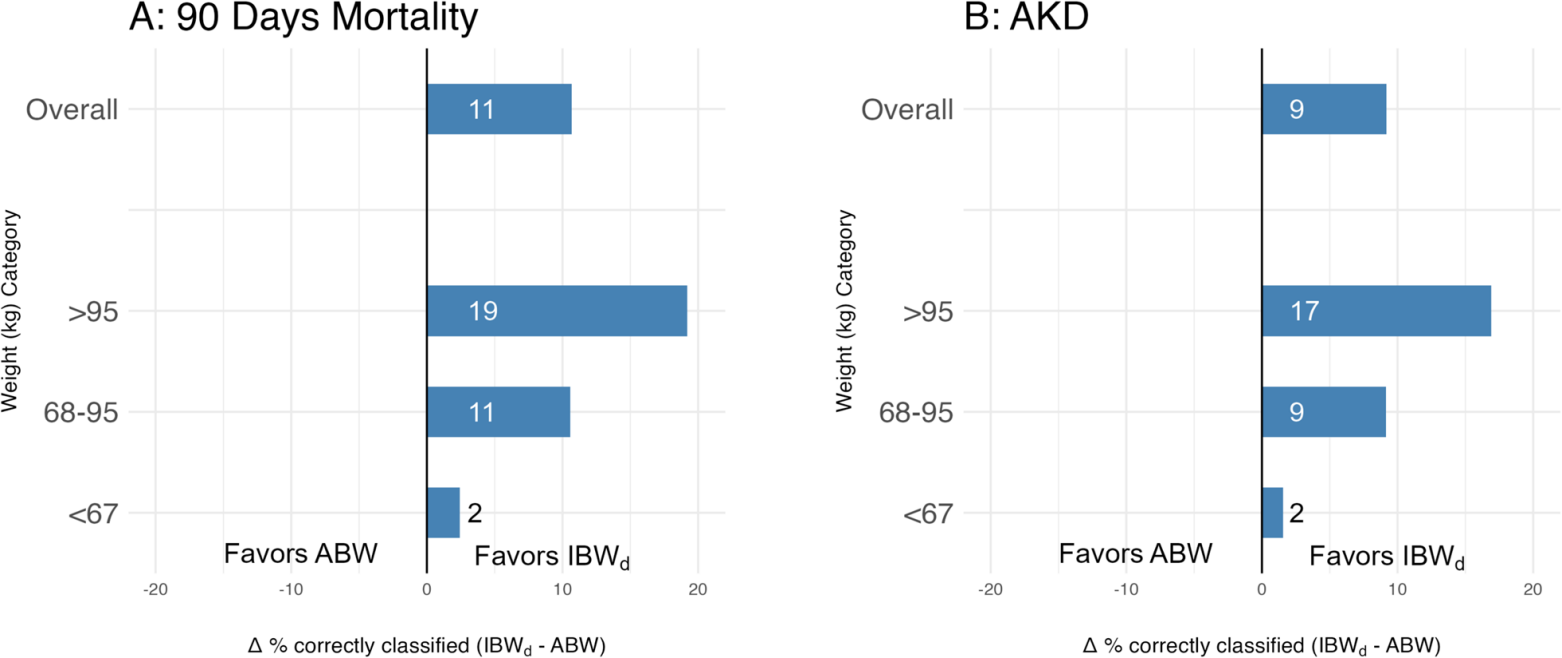
Difference in the proportion of patients correctly classified by type of UO normalization. Panel A: 90-day mortality (correct classification = patients with oliguria who were dead at day 90 AND patients without oliguria who were alive at day 90). Panel B: Acute Kidney Disease (correct classification = patients with oliguria who had AKD at hospital discharge AND patients without oliguria who did not have AKD at hospital discharge). ABW: actual body weight; IBWd: ideal body weight; UO: urine output; AKD: acute kidney disease

As shown in Table 2, compared with ABW, normalization of UO by IBW_d_ increased the strength of the association between oliguria and 90-day mortality (pseudo R^2^ 4.4 versus 2.1) and AKD (pseudo R^2^ 2.5 versus 1.7).This persisted after correction for sex and corrected SAPS-II score (Table S4). As shown in Fig. 3, this translates into a higher proportion of patients with a correct prediction of both mortality and AKD when IBW_d_ is considered for UO normalization, compared with ABW (mortality: 48.3 versus 37.7%, AKD 47.0 versus 37.8%; details in Table S5). This was particularly striking for patients with high (> 95 kg) ABW.

**Table 2:** Association between oliguria and outcomes according to the different types of normalization (and sensitivity analyses)

UO normalized by	90-day mortality		AKD	
	AUC (95%CI)	Pseudo R^2^	AUC (95%CI)	Pseudo R^2^
All patients (n = 28 610)				
ABW	0.57 (0.57,0.58)	2.14	0.57 (0.56,0.57)	1.65
IBW_d_	0.62 (0.62,0.63)	4.45	0.59 (0.59,0.60)	2.47
Patients with an indwelling catheter throughout icu stay (n = 17 284)				
ABW	0.57 (0.56,0.58)	2.04	0.56 (0.55,0.57)	1.43
IBW_d_	0.63 (0.62,0.63)	5.07	0.59 (0.58,0.60)	2.56
Patients who never received diuretics (n = 12 677)				
ABW	0.60 (0.59,0.61)	3.26	0.58 (0.57,0.59)	1.63
IBW_d_	0.65 (0.64,0.66)	5.64	0.60 (0.58,0.61)	2.44

AKD, acute kidney disease at hospital discharge; AUC, area under the receiver operator curve; ABW, actual body weight; IBWd, ideal body weight; patients with free micturitions: patients who did not have an indwelling catheter for the entire duration of their icu stay (free micturitions)

### Overlap and agreement between UO and sCr criteria

The maximum AKI stage observed using sCr or UO criteria and their overlap are presented in Table S6. The agreement between the two methods was poor but marginally better when UO was normalized by IBW_d_ rather than by ABW (Kendall correlation coefficient 0.35 versus 0.34). Similar AKI stage using sCr or UO was reached in a higher proportion of patients when UO was normalized by IBW_d_ than with ABW (40.8 versus 30.7%).

### Sensitivity analyzes

We repeated our analyses in the validation cohort considering only patients with an indwelling catheter for the entire duration of their ICU stay (n = 17 284) and those who never received diuretics during ICU stay (n = 12 677), before (Table 2) and after correction for SAPS-II and sex (Table S4). These analyses largely confirmed our main results.

## Discussion

### Key findings

We performed an observational study in almost 45 000 patients from two different countries, with very different weight distributions. We found that, among a large set of candidate variables (including ABW, IBW, BMI, BSA or adjBW), height derived parameters (IBW) were the strongest predictors of patients’ UO during their ICU stay. We found that UO normalization by ABW lead to an almost linear increase in the observed incidence of oliguria across weight categories and an overestimation of oliguria, particularly in patients with high ABW. We found that normalization of UO by IBW_d_ rather than ABW led to a more stable incidence of oliguria and an increased strength of its association with outcomes. These findings were confirmed in the validation cohort and remained after adjustment for confounding factors.

### Comparison with previous studies

The transition from absolute values to weight-standardized thresholds to define oliguria has arisen in the pediatric literature before being translated into the adult literature [25–27]. Although such an adjustment appears logical, the supporting evidence remains, however, limited. To the best of our knowledge, our study is the first to describe the relationship between height, weight and patient’s minimal, maximal and mean 6-h UO during their ICU stay. We found that patients’ UO was correlated to height or height-derived parameters (IBWs) rather than weight-derived parameters (BMI, BSA, ABW, adjBW) suggesting that using an IBW to normalize UO may be more appropriate.

We further noted that the normalization method influenced the observed incidence of oliguria and that normalization by ABW lead to a higher observed incidence than normalization with the IBW_d_ (71.7 versus 66.3%). This finding is consistent with previous works [8–11]. However, the observation of a nearly linear relationship between oliguria incidence and categories of BW is new. Hence, normalization by ABW might lead to an overestimation of the incidence of oliguria, particularly in obese patients. Moreover, previous data were mostly derived from small single-center retrospective studies [8, 11], specific populations (sepsis and cardiac surgery patients) [10, 11] or limited to a single country with a specific weight distribution [9]. Also, some used an adjBW as a surrogate for IBW in obese patients [8, 9]. Our data suggests that, compared to adjBW, IBW was more closely associated with UO during the ICU stay and performed better to predict outcomes. In addition, the incidence of oliguria was higher in our study than in previous publications, probably due to a very strict application of UO criteria.

Altogether, our results are in agreement with most of the earlier studies showing a better association between oliguria and poor outcomes when normalizing UO with the IBW rather than the ABW [8–10]. Only one small single-center retrospective study failed to replicate this finding. However, in this cohort, patients’ ABW and IBW were nearly identical (59 *vs.* 58 kg) explaining the observed lack of difference between the two methods of normalization [11]. Furthermore, while previous studies mainly focused on in-hospital [9, 10] or 90-day mortality [8, 9], we also demonstrated an improved association between oliguria and AKD at hospital discharge when normalizing with IBW_d_ instead of ABW, particularly in obese patients.

### Implications for clinicians, researchers and policy makers

Our results strongly suggest that IBW should be used to normalize UO. This finding is highly relevant to both research and routine clinical practice. Normalization with ABW may overestimate the incidence of AKI particularly in obese patients. This might lead to unwarranted investigations and potentially harmful therapeutic interventions such as fluid or diuretic administration when not required [28, 29]. Furthermore, height appears to be a more reliable parameter than weight as it is easy to obtain in unconscious patients and it is neither subject to variation nor influenced by fluid overload or muscle mass loss, both common issues in critically ill patients. Previous studies have clearly shown that weight estimations in ICU are often inaccurate, in contrast to height estimations[5, 6]. IBW is already widely used in the ICU as this parameter serves as the reference to adjust tidal volume for protective ventilation. Additionally, the generalization of IBW to normalize UO would lead to a more uniform application of KDIGO criteria and improve the comparability between studies and centers. Therefore, we suggest that future iterations of AKI diagnostic criteria recommend to normalize UO by IBW.

### Strengths and limitations

This study has several strengths. First, it is the largest to assess the impact of the normalization method for UO. It leverages two datasets, from two different countries and healthcare systems. The datasets presented major differences in patients types and weights distributions reinforcing our results and improving their generalizability. Second, we tested a large panel of candidate variables to identify the best predictor of UO during ICU stay. Our data strongly suggest that UO would more closely be related to height rather than weight. Third, the selected predictor has the advantage of being universally available in critically ill patients, to be commonly utilized in ICU (to adjust tidal volume in mechanically ventilated patients) and, to represent a minor change from the currently utilized parameter (as despite being a function of size, it remains called “weight”). Fourth, our results were consistent across the two datasets, persisted after correction for major confounders in multivariate analyses and in sensitivity analyses.

This study also has limitations worth discussing. First, ABW, our reference weight, is known to be highly imprecise in critically ill patients. In particular, it may have been overestimated due to fluid overload, which is very common in critically ill patients. In order to minimize this bias, we have considered the preadmission weight whenever available in the derivation cohort (79% of patients) and in the validation cohort we included only patients with available pre admission weight. However, the difficulty of obtaining reliable BW estimation actually further reinforces our findings that size-based measurements should be preferred. Second, diuretic use was not considered in our analyses and could have biased the evaluation of oliguria incidence. However, a sensitivity analysis excluding patients who received diuretics at any stage during their ICU stay did not modify our results. Third, AKD at hospital discharge is not a recognized endpoint and might have been biased by a loss of lean mass associated with critical illness [30]. This effect may be even more pronounced in obese patients, known to have a higher absolute muscle mass [31]. However, this would lead to an underestimation of AKD at hospital discharge decreasing the likelihood of observing a difference between the two methods.

## Conclusions

In this large cohort study, we found that, among a large set of candidate variables, IBW was the best predictor of UO during ICU stay. UO normalization by IBW_d_ lead to a stable incidence of oliguria across categories of BW and appeared more closely associated with outcomes. IBW_d_ should be preferred to normalize UO in critically ill patients in ICU protocols and future guidelines iterations.

## Supplementary Information

Supplementary Material 1.

## Data Availability

The datasets used and/or analyzed as well as R code used in the current study are available from the corresponding author upon reasonable request.

## Abbreviations

ABW: Actual body weight
AKD: Acute kidney disease
AKI: Acute kidney injury
KDIGO: Kidney disease: improving global outcomes
IBW: Ideal body weight
ICU: Intensive care unit
sCr: Serum creatinine
STROBE: Strengthening the reporting of observational studies in epidemiology
UO: Urine output

## Declarations

**Ethical approval and consent to participate**

The study protocol was approved by the Ethics Committee of Vaud, Switzerland (CER-VD 2017–00008).
