# Supplementary material for: Comparison of methods to normalize urine output in critically ill patients: a multicenter cohort study
Source: Crit Care. 2024 Dec 19;28:425. doi: 10.1186/s13054-024-05200-x (PMC11658224; doi:10.1186/s13054-024-05200-x)
Supplement: Supplementary file 1 — Supplementary Material 1. [file 13054_2024_5200_MOESM1_ESM.docx]

Supplementary material for

# Comparison of Methods to Normalize Urine Output in Critically Ill Patients: a Multicenter Cohort Study

**Authors:** Céline Monard, Nicolas Tebib, Bastien Trächsel, Tatiana Kelevina, Antoine Guillaume Schneider

**Corresponding Author:**

Antoine Schneider

Adult Intensive Care Unit,

Centre Hospitalier Universitaire Vaudois,

1011 Lausanne, Switzerland.

E-mail: antoine.schneider@chuv.ch

Table of contents

[Comparison of Methods to Normalize Urine Output in Critically Ill Patients: a Multicenter Cohort Study 1](#_Toc182848927)

[Figure S1- Patients’ flow chart in the validation and the derivation cohort. 2](#_Toc182848928)

[Figure S2 –Association between **minimal** urine output (UO) over 6 hours during ICU stay and actual body weight (panel A) or height (panel B) in critically ill patients. 3](#_Toc182848929)

[Figure S3 –Association between **maximal** urine output (UO) over 6 hours during ICU stay and actual body weight (panel A) or height (panel B) in critically ill patients 4](#_Toc182848930)

[Table S1- Differences between the derivation and validation cohort 5](#_Toc182848931)

[Table S2- Formulas for candidate variables calculations. 6](#_Toc182848932)

[Table S3- Accuracy of different candidate variables to predict the mean 6-jours urine output during ICU stay. 7](#_Toc182848933)

[Table S4- Association between oliguria and outcomes according to the different types of normalization adjusted for sex, and SAPS-II 8](#_Toc182848934)

[Table S5: Number (percentage) of patients correctly classified for 90-day mortality and acute kidney disease, according to the variable used for UO normalization and the weight category. 9](#_Toc182848935)

[Table S6- Agreement between UO and sCr criteria for AKI diagnosis and staging according to UO normalization 10](#_Toc182848936)

# Figure S1- Patients’ flow chart in the validation and the derivation cohort.


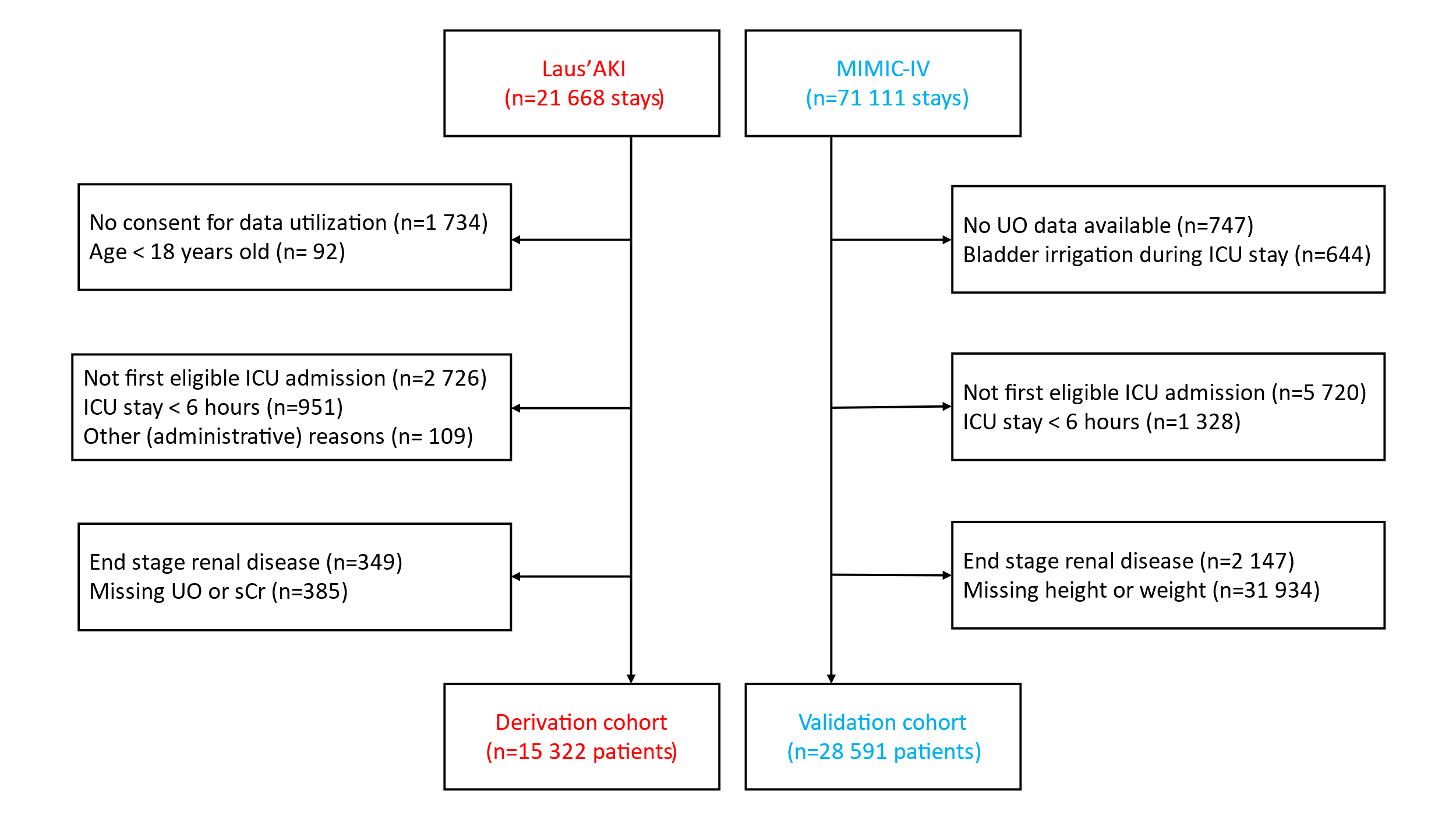


# Figure S2 –Association between **minimal** urine output (UO) over 6 hours during ICU stay and actual body weight (panel A) or height (panel B) in critically ill patients.


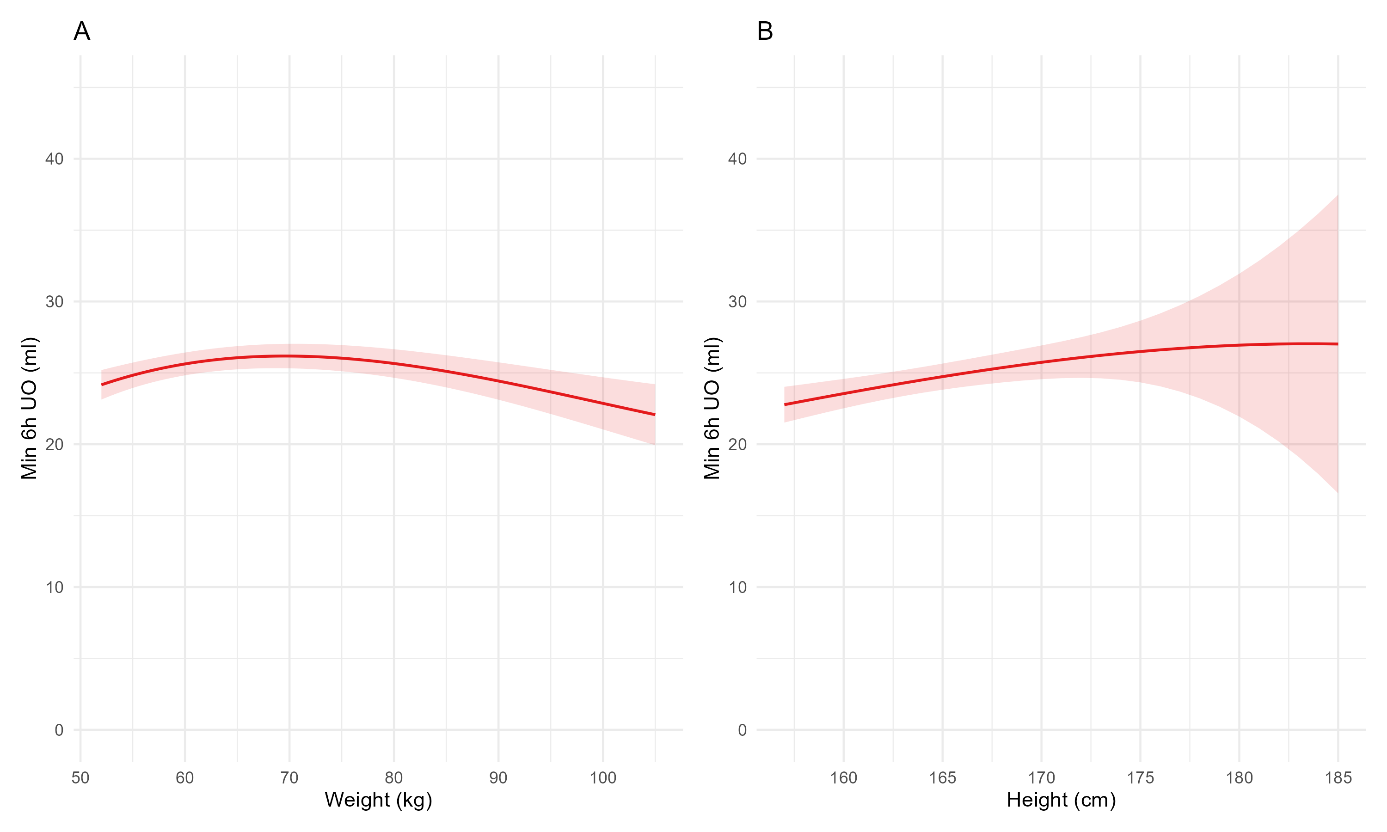


# Figure S3 –Association between **maximal** urine output (UO) over 6 hours during ICU stay and actual body weight (panel A) or height (panel B) in critically ill patients


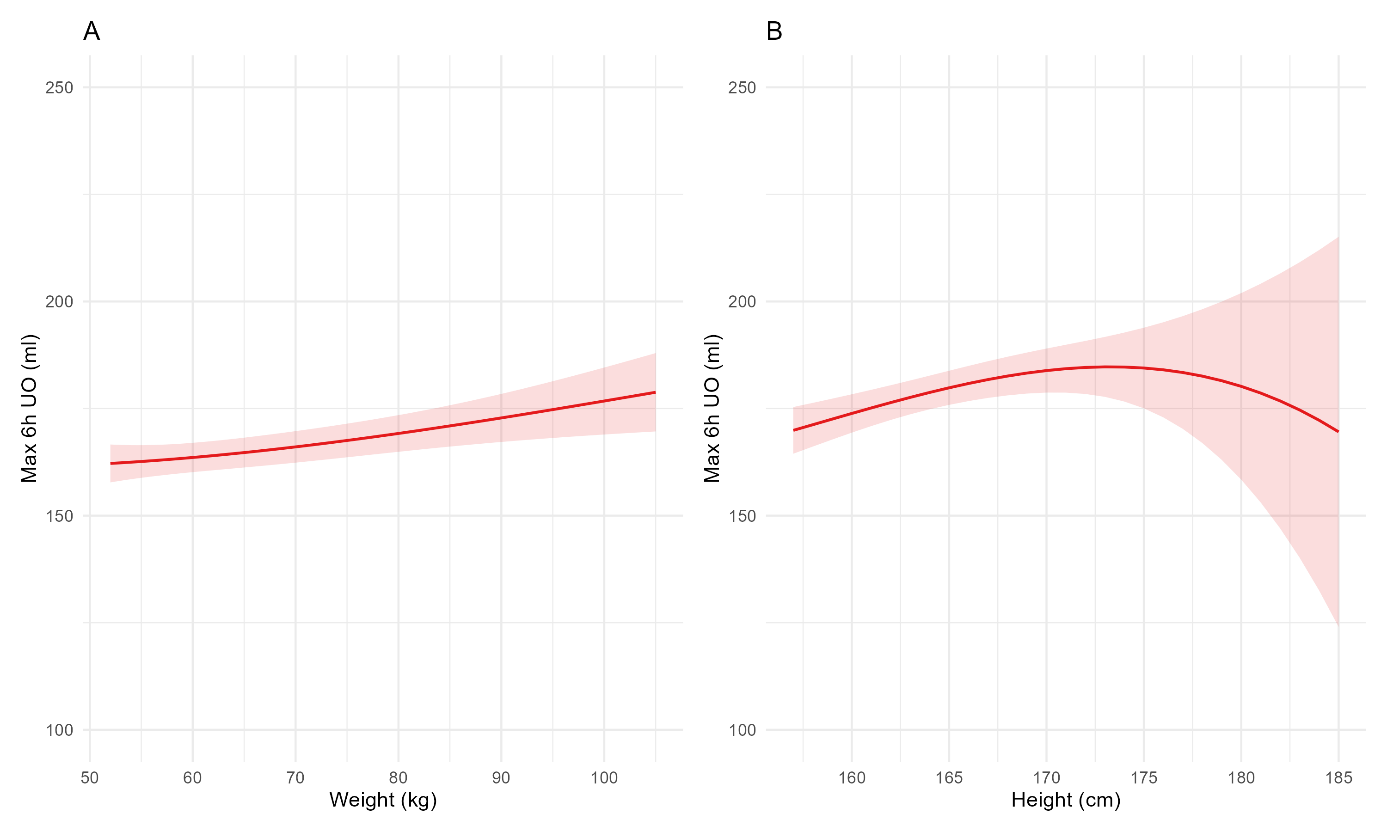


# Table S1- Differences between the derivation and validation cohort

|  | Derivation cohort  (Laus’AKI) | Validation cohort  (MIMIC-IV) |
| --- | --- | --- |
| Population selection | | |
| End stage renal disease (ESRD) criteria | Patients receiving maintenance dialysis only | Those with ESRD and/or receiving maintenance dialysis |
| Stays without sCr measurement during stay | Excluded | Included |
| Other |  | Exclusion of patients who received at least one vesical irrigation during ICU stay |
| Oliguria assessment | | |
| UO values measured during RRT | Considered | Not considered, replaced by missing values |
| Abnormal UO values | Hourly UO value greater than 1'000 ml after processing of missing values were considered as erroneous and replaced by the closest hourly UO value available | Hourly UO value greater than 2’500ml have been removed.  (42 measurements out of 3’250’174) |
| Actual body weight definition | The considered body weight is the value recorded at ICU admission. When unknown, the first quartile of all body weights measured during ICU stay is considered. If no body weights were measured during ICU stay, 60kg was assumed for women and 70kg for men. | Value available in the MIMIC-IV cohort under “Admission Weight (Kg)” in chart events |
| Factors’ definitions | | |
| Baseline sCr | The lowest sCr within the 12 months before ICU admission at the exception of those before an emergency admission; if such value is not available, consideration of the lowest value during ICU stay. | If the lowest sCr value during ICU stay is normal, it was considered.  Otherwise:   1. If the patient was diagnosed with CKD, then the lowest sCr value during the stay was considered 2. If the patient was **not** diagnosed with CKD, the baseline sCr was calculated using the Simplified MDRD Formula and assuming an eGFR of 75 |
| sCr: serum creatinine; UO: urine output; CKD: chronic kidney disease | | |

# Table S2- Formulas for candidate variables calculations.

|  | |
| --- | --- |
| Formula | **Equation** |
| IBW | |
| Devine formula (IBW_Devine_)  Male  Female | 50 kg + 0.9 kg × (height (cm) – 152)  45.5 kg + 0.9 kg × (height (cm) − 152) |
| Peterson formula | 2.2 × BMI + 3.5 × BMI × (height (cm) − 1.5 m) |
| Hammond formula  Male  Female | 48 kg + 1.1 kg × (height (cm) − 150 cm)  45 kg + 0.9 kg × (height (cm) − 150 cm) |
| Miller formula  Male  Female | 56.2 kg + 1.41 kg × (height (cm) – 152)  53.1 kg + 1.36 kg × (height (cm) − 152) |
| AdjBW | IBW_Devine_ + 0.4 x (ABW- IBW_Devine_) |
| BMI | weight (kg)/height^2^ (m) |
| BSA |  |
| Mosteller formula | 0.016667 x weight (kg)^0.5^ x height (cm)^0.5^ |
| Dubois formula | 0.007184 x weight (kg)^0.425^ x height (cm)^0.725^ |
| Haycock formula | 0.024265 x weight (kg)^0.5378^ x height (cm)^0.3964^ |
| IBW: ideal body weight, AdjBW: adjusted body weight; ABW: actual body weight, BMI: body mass index, BSA: body surface area | |

# Table S3- Accuracy of different candidate variables to predict the mean 6-hours urine output during ICU stay.

| Variable tested | AIC | R^2^ |
| --- | --- | --- |
| ABW | 156837.2 | 0.47 |
| IBW | | |
| Devine formula (IBW_d_) | 127273.3 | 0.9 |
| Peterson formula | 127318.2 | 0.54 |
| Hammond formula | 127276.1 | 0.88 |
| Miller formula | 127275.2 | 0.88 |
| AdjBW | 127274.7 | 0.89 |
| Height | 127265.6 | 0.96 |
| BMI | 127368.6 | 0.14 |
| BSA |  |  |
| Mosteller formula | 127298.5 | 0.7 |
| Dubois formula | 127288.5 | 0.78 |
| Haycock formula | 127302.9 | 0.66 |
| AIC: Akaike information criteria; R^2^: R-squared, IBW: ideal body weight, AdjBW: adjusted body weight; ABW: actual body weight, BMI: body mass index, BSA: body surface area  For AIC, the lowest value indicates the best performance  For R^2^, the highest value indicates the best performance | | |

AIC values compare models considering both the goodness of fit and the number of parameters used, with lower values indicating a better model. The R^2^ represents the percentage of variation explained by the model; a R^2^ of 100 would mean that we could perfectly predict all deaths with the variables we have in the model, while an R^2^ of 0 means that the variables are not useful.

# Table S4- Association between oliguria and outcomes according to the different types of normalization adjusted for sex, and SAPS-II

|  | 90-day mortality | | AKD | |
| --- | --- | --- | --- | --- |
|  | AUC (95%CI) | Pseudo R^2^ | AUC (95%CI) | Pseudo R^2^ |
| All patients (n=28 591) |  |  |  |  |
| UO normalized by ABW | 0.74 (0.74,0.75) | 12.07 | 0.67 (0.66,0.68) | 5.67 |
| UO normalized by IBW_d_ | 0.76 (0.75,0.76) | 13.53 | 0.68 (0.67,0.68) | 6.22 |
| Patients with an indwelling catheter throughout ICU stay (n=17 284) | | | |  |
| UO normalized by ABW | 0.73 (0.72,0.74) | 11.34 | 0.66 (0.65,0.67) | 4.93 |
| UO normalized by IBW_d_ | 0.75 (0.74,0.76) | 13.37 | 0.67 (0.66,0.68) | 5.72 |
| Patients who never received diuretics (n=12 677) | | | |  |
| UO normalized by ABW | 0.81 (0.8,0.82) | 21.66 | 0.69 (0.68,0.71) | 7.9 |
| UO normalized by IBW_d_ | 0.82 (0.81,0.83) | 22.83 | 0.7 (0.69,0.71) | 8.28 |
| AKD: Acute Kidney Disease at hospital discharge, IBW_d_ : ideal body weight, ABW: actual body weight, AUC: area under the ROC curve | | | | |

The results for AUC are AUC (95%CI), and the pseudo R^2^ represents the percentage of variation explained by the model; an R^2^ of 100 would mean that we could perfectly predict all deaths with the variables we have in the model, while an R^2^ of 0 means that the variables are not useful.

# Table S5- Number (percentage) of patients correctly classified for 90-day mortality and acute kidney disease, according to the variable used for UO normalization and the weight category.

| Outcome | UO normalization by: | **Low weight** | **Normal weight** | **High weight** | **All** |
| --- | --- | --- | --- | --- | --- |
| **90-day mortality** | **ABW** | 3 595 (51.1%) | 5 431 (37.3%) | 1 749 (25%) | 10 775 (37.7%) |
|  | **IBW_d_** | 3 765 (53.5%) | 6 968 (47.9%) | 3 094 (44.2%) | 13 827 (48.4%) |
| **AKD** | **ABW** | 3 199 (45.5%) | 5 513 (37.9%) | 2 100 (30%) | 10 812 (37.8%) |
|  | **IBW_d_** | 3 309 (47%) | 6 843 (47%) | 3 282 (46.9%) | 13 434 (47%) |
| AKD: Acute Kidney Disease at hospital discharge, IBW_d_ : ideal body weight, ABW: actual body weight, Low weight: <67kg of ABW, Normal weight 68-95kg of ABW, High weight >95kg of ABW; N= 28 591 | | | | | |

# Table S6- Agreement between UO and sCr criteria for AKI diagnosis and staging according to UO normalization

|  |  |  | Max sCr stage | | | |  |
| --- | --- | --- | --- | --- | --- | --- | --- |
|  |  |  | No AKI | Stage 1 | Stage 2 | Stage 3 | Agree-ment |
| Max UO stage | UO normalized  by ABW | No AKI | **5665** | 856 | 163 | 66 | 0.34 |
|  |  | Stage 1 | 3954 | **1109** | 199 | 93 |  |
|  |  | Stage 2 | 7045 | 3315 | **816** | 445 |  |
|  |  | Stage 3 | 1404 | 1480 | 764 | **1176** |  |
|  | UO normalized by IBW_D_ | No AKI | **8307** | 1574 | 276 | 114 | 0.35 |
|  |  | Stage 1 | 4120 | **1463** | 284 | 120 |  |
|  |  | Stage 2 | 4830 | 2841 | **843** | 521 |  |
|  |  | Stage 3 | 811 | 882 | 539 | **1025** |  |
| N= 28 550. Bold numbers correspond to patients who reached similar AKI stage with both UO or sCr criteria (11 638 (40.8%) with IBW_D_ normalization and 8 766 (30.7%) with ABW normalization). Agreement (Kendall correlation coefficient).  sCr: serum creatinine, UO: urine output, ABW: actual body weight, IBW_D:_ ideal body weight defined by the Devine formula. | | | | | | | |
